# Synthesis and Characterization of Citrate-Stabilized Gold-Coated Superparamagnetic Iron Oxide Nanoparticles for Biomedical Applications

**DOI:** 10.3390/molecules25194425

**Published:** 2020-09-26

**Authors:** René Stein, Bernhard Friedrich, Marina Mühlberger, Nadine Cebulla, Eveline Schreiber, Rainer Tietze, Iwona Cicha, Christoph Alexiou, Silvio Dutz, Aldo R. Boccaccini, Harald Unterweger

**Affiliations:** 1 Department of Otorhinolaryngology-Head and Neck Surgery, Section of Experimental Oncology and Nanomedicine (SEON), Else Kroener-Fresenius-Stiftung-Professorship, Universitätsklinikum, 91054 Erlangen, Germany; bernhard.friedrich@uk-erlangen.de (B.F.); marinamuehlberger@googlemail.com (M.M.); nadinecebulla@gmx.de (N.C.); eveline.schreiber@uk-erlangen.de (E.S.); rainer.tietze@uk-erlangen.de (R.T.); iwona.cicha@uk-erlangen.de (I.C.); Christoph.Alexiou@uk-erlangen.de (C.A.); 2 Institute of Biomedical Engineering and Informatics, Technische Universität Ilmenau, 98693 Ilmenau, Germany; Silvio.Dutz@tu-ilmenau.de; 3 Institute of Biomaterials, University of Erlangen-Nuremberg, 91058 Erlangen, Germany; aldo.boccaccini@ww.uni-erlangen.de

**Keywords:** nanoparticles, superparamagnetic iron oxide nanoparticles (SPIONs), gold coating, thiol-binding, surface functionalization, characterization, cytotoxicity

## Abstract

Surface-functionalized gold-coated superparamagnetic iron oxide nanoparticles (Au-SPIONs) may be a useful tool in various biomedical applications. To obtain Au-SPIONs, gold salt was precipitated onto citrate-stabilized SPIONs (Cit-SPIONs) using a simple, aqueous one-pot technique inspired by the Turkevich method of gold nanoparticle synthesis. By the further stabilization of the Au-SPION surface with additional citrate (Cit-Au-SPIONs), controllable and reproducible Z-averages enhanced long-term dispersion stability and moderate dispersion pH values were achieved. The citrate concentration of the reaction solution and the gold/iron ratio was found to have a major influence on the particle characteristics. While the gold-coating reduced the saturation magnetization to 40.7% in comparison to pure Cit-SPIONs, the superparamagnetic behavior of Cit-Au-SPIONs was maintained. The formation of nanosized gold on the SPION surface was confirmed by X-ray diffraction measurements. Cit-Au-SPION concentrations of up to 100 µg Fe/mL for 48 h had no cytotoxic effect on Jurkat cells. At a particle concentration of 100 µg Fe/mL, Jurkat cells were found to take up Cit-Au-SPIONs after 24 h of incubation. A significantly higher attachment of thiol-containing L-cysteine to the particle surface was observed for Cit-Au-SPIONs (53%) in comparison to pure Cit-SPIONs (7%).

## 1. Introduction

Gold nanoparticles (Au-NPs) or gold-coated nanoparticles have extraordinary material properties that make them particularly interesting for biomedical applications. In particular, the surface-binding property of gold gives rise to a wide variety of possible utilizations. Simple and strong binding motifs on Au-NPs are found, e.g., for thiols or disulfide compounds [1,2,3]. Selenides, isothiocyanates and phosphines also show a stable surface attachment. In addition, amines and carboxylates exhibit an affinity with the gold surface as well [4]. The functionalization of gold surfaces is far from being fully understood and novel functionalization strategies are still being discovered that lead to very stable and selective linkers, as recently described by De Jesus et al. in the form of new N-heterocyclic carbenes [5].

In applications as bioprobes, gold surfaces can be functionalized with antibodies or DNA aptamers, thus binding the corresponding complementary molecules. For the subsequent detection of these interactions, the enzyme-mediated growth or aggregation of Au-NPs is employed [6]. In the same way, components of viruses can be bound as antigens to gold nanoparticles to develop improved vaccination strategies [7]. Furthermore, surface modifications can be used, e.g., to create drug delivery systems when functionalized with therapeutics [8,9,10,11], as well as for photothermal therapy for cancer [12,13,14,15]. The biodistribution of pure Au-NPs is, however, hardly controllable, which hampers the accumulation of the particles in the target tissue and therefore their efficacy during application [16,17,18].

Due to their magnetic properties, superparamagnetic iron oxide nanoparticles (SPIONs) are likewise promising for biomedical applications. Modification of their surface enables SPIONs to be used, e.g., as contrast agents in magnetic resonance imaging [19,20] or as drug delivery systems for the magnetic drug targeting of tumors and vascular injuries [21,22,23]. By combining the highly magnetic properties of SPIONs with an easy-to-functionalize gold surface, a fundamental hybrid carrier system (Au-SPIONs) can be generated [24,25,26,27], on which molecules designed for various medical applications can be attached. Hence, applications such as photothermal or drug delivery therapy may not be limited by the unspecific accumulation of the particles in the tissue as Au-SPIONs can be magnetically guided for a control of their biodistribution and localization of their efficacy in the targeted tissue. Furthermore, Au-SPIONs may exhibit enhanced biocompatibility in comparison to mere SPIONs due to their bioinert gold surface.

The functionalization and corresponding biomedical application can only be successful with a simple, reliable and reproducible aqueous synthesis that delivers nanoparticles with the appropriate qualities. Many of the procedures found in the literature, however, are based on non-aqueous SPION synthesis; e.g., thermal decomposition or micro-emulsion methods [24,28,29,30,31,32], which require potentially harmful substances or solvents in steps of their synthesis. For aqueous and non-harmful procedures [27,33,34,35], no studies were published, which shows the systematic optimization of the synthesis parameters and the evaluation of their effects on Au-SPION properties such as particle size, dispersion stability and reproducibility.

By modifying and adapting the method of Elbialy et al. [34], we present in this study a simple, aqueous synthesis procedure for a highly reproducible and size controllable production of citrate-stabilized gold-coated SPIONs (Cit-Au-SPIONs). This research highly focuses on providing an understanding for the effects of various synthesis parameters on the produced nanoparticles as they are systematically investigated. The synthesized particles are physicochemically characterized and further tested for cytotoxicity to ensure biocompatibility. As a proof of concept, the thiol group containing amino acid L-cysteine is attached to the citrate-stabilized gold surface to show the facile thiol–gold binding motif and surface functionalization of Cit-Au-SPIONs, which may be used in various medical purposes.

## 2. Results

### 2.1. Characterization of Cit-SPIONs, Au-SPIONs and Cit-Au-SPIONs

**Figure 1:**
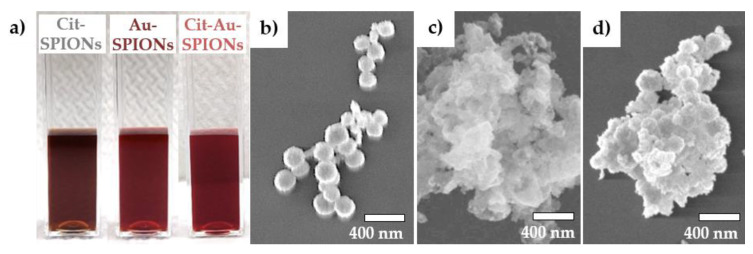
(**a**) Particle dispersions of pure citrate-stabilized (Cit)-superparamagnetic iron oxide nanoparticles (SPIONs) (left), Au-SPIONs (middle) and Cit-Au-SPIONs (right) as well as scanning electron microscopy (SEM) images of (**b**) pure Cit-SPION clusters, (**c**) Au-SPION clusters and (**d**) Cit-Au-SPION clusters.

**Figure 2:**
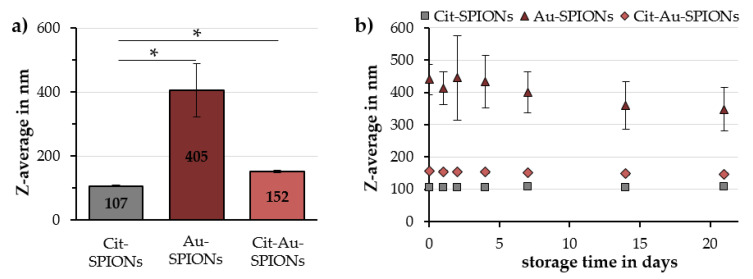
(**a**) Mean Z-averages over 21 days for Cit-SPIONs, Au-SPIONs and Cit-Au-SPIONs; (**b**) Z-Averages of Cit-SPIONs, Au-SPIONs and Cit-Au-SPIONs during storage for different time points. Statistically significant changes are marked with * for *p* < 0.05.

**Figure A1:**
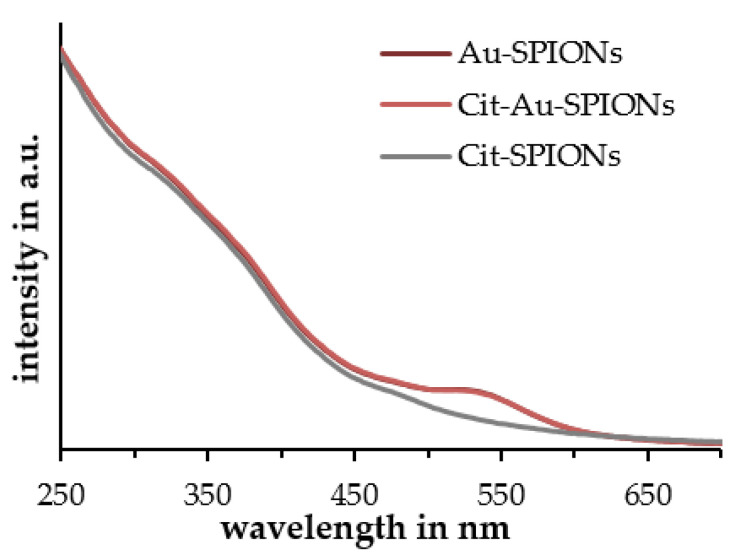
UV–Vis measurements of Cit-SPIONs, Au-SPIONs and Cit-Au-SPIONs showing an intensity peak at a wavelength of 520 nm for both gold-coated SPION independent of additional citrate-stabilization.

**Figure A2:**
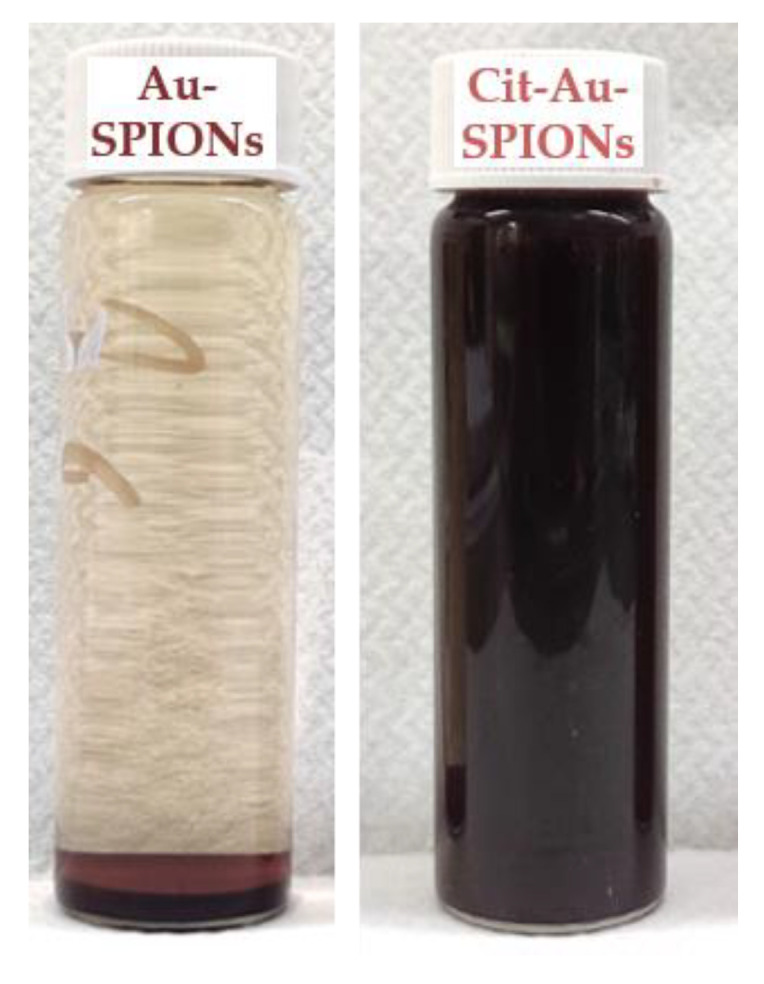
Dispersion stability of Au-SPIONs and Cit-Au-SPIONs after one week of storage at 4 °C.

During the reduction of gold onto Cit-SPIONs to generate SPION cores with a surrounding gold shell, the color of the particle dispersion changed from black to a deep wine red, as seen in Figure 1a. There were no major differences in color between non-stabilized Au-SPIONs and Cit-Au-SPIONs, which have additional citrate on their gold shell. This was further confirmed by ultraviolet–visible spectroscopy measurements, which are shown in Figure A1. On scanning electron microscopy (SEM) images, Cit-Au-SPION clusters (Figure 1d) resemble the spherical clusters seen in pure Cit-SPIONs (Figure 1b), whereas non-stabilized Au-SPIONs form large irregularly shaped structures (Figure 1c). Accordingly, dynamic light scattering (DLS) data (Figure 2a) display a large increase in the Z-average from 107 ± 3 nm with a corresponding polydispersity index (PDI) of 0.15 ± 0.03 in pure Cit-SPIONs to 405 ± 83 nm with a corresponding PDI of 0.25 ± 0.06 in non-stabilized Au-SPIONs. As shown in Figure 2b, a huge deviation in the Z-average was observed between different Au-SPION batches as well as during the storage for 21 days. The presence of additional citrate ions on Cit-Au-SPIONs reduced the Z-average to 152 ± 5 nm with a corresponding PDI of 0.19 ± 0.01, improved the reproducibility in size of different particle batches and enhanced the long-term stability during storage. Cit-Au-SPIONs exhibited similar storage stability as pure Cit-SPIONs, with only small changes in their Z-averages over 21 days. In contrast, non-stabilized Au-SPIONs sedimented out of dispersion after one week of storage at 4 °C, as shown in Figure A2.

**Figure A3:**
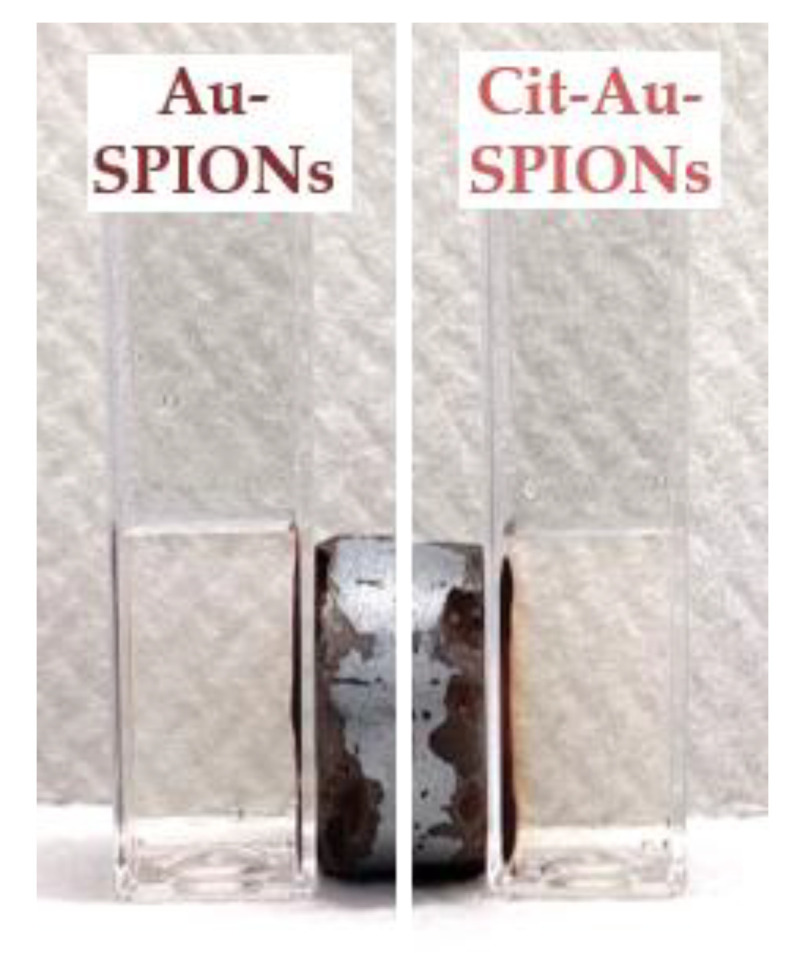
Magnetic separation of Au-SPIONs and Cit-Au-SPIONs after 8 h showed clear supernatants, which indicated that the gold was reduced onto the SPION cores instead of forming unattached nanosized gold that would appear red in the supernatant.

To investigate whether the SPIONs were coated with gold or whether pure gold nanoparticles formed without any attachment to the SPIONs, a magnet was placed next to the Au-SPION and Cit-Au-SPION dispersions. The supernatants of both samples were colorless after 8 h, which indicated that any nanosized gold is attached to the SPIONs and can be manipulated using a magnet (Figure A3). At an adjusted pH of 7.0, Cit-SPIONs, Au-SPIONs and Cit-Au-SPIONs exhibit a ζ-potential of −48.0 ± 6.3, −43.5 ± 0.6 and −48.6 ± 0.3 mV, respectively, as listed in Table 1. The pH values of the particle dispersions after synthesis were 8.27 ± 0.07, 2.89 ± 0.14 and 6.21 ± 0.14 for Cit SPIONs, Au-SPIONs and Cit-Au-SPIONs, respectively.

**Figure 3:**
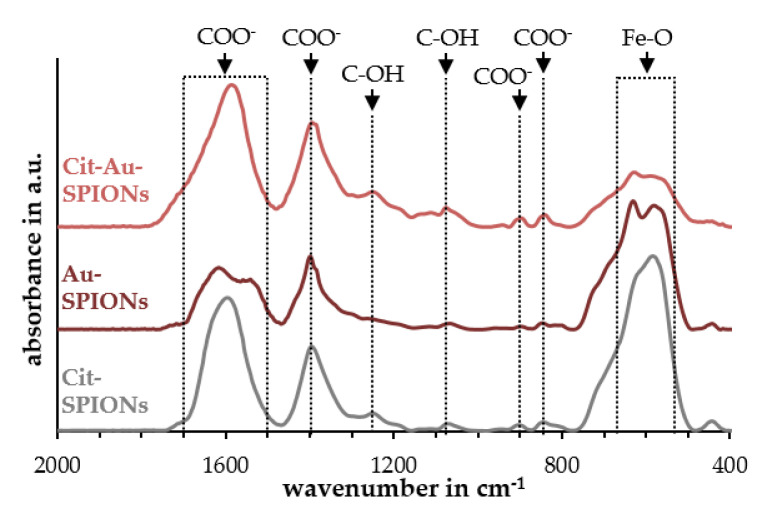
FTIR spectra of Cit-SPIONs, Au-SPIONs and Cit-Au-SPIONs. Peak identification was performed using [36,37,38,39,40].

Fourier-transform infrared spectroscopy (FTIR) measurements, shown in Figure 3, were used to investigate the citrate stabilization on the particles. Cit-SPIONs, Au-SPIONs and Cit-Au-SPIONs all showed a characteristic iron-oxygen (Fe-O) band between 550 and 650 cm^−1^ [36,37]. Characteristic peaks of deprotonated carboxylic acid groups (COO^−^) at ~844, ~904 and 1397 cm^−1^, as well as a broad band between 1550 and 1750 cm^−1^, were found in all three particle samples [38,39,40]. Peaks that could be related to hydroxyl groups (C-OH) oscillations at ~1070 and ~1275 cm^−1^ [38,39,40] were absent, or clearly less pronounced, in the spectrum of non-stabilized Au-SPIONs when compared to the spectra of Cit-SPIONs and Cit-Au-SPIONs. Furthermore, the relative intensities between the Fe-O band and the COO^−^ features in the Au-SPION spectrum were lower in comparison to the relative intensities of the features in the Cit-SPION and in the Cit-Au-SPION spectrum. As an example, the I_COO_/I_Fe-O_ ratio for the 904 cm^−1^ COO^−^ peak was calculated to be 0.039, 0.025 and 0.166 for Cit-SPION, Au-SPIONs and Cit-Au-SPIONs, respectively.

**Figure 4:**
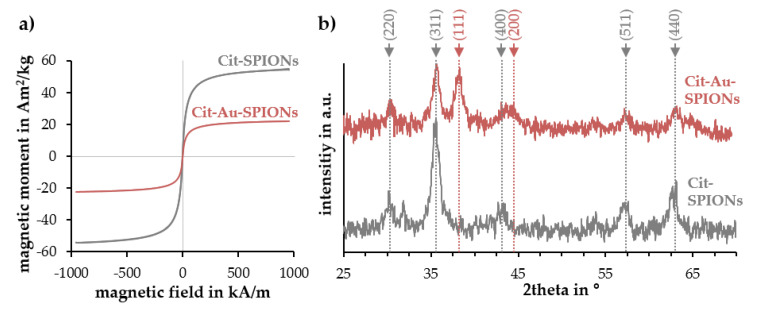
(**a**) Magnetization curves and (**b**) XRD measurements of Cit-SPIONs and Cit-Au-SPIONs. Peak identification was performed using [41,42].

Due to the enhanced reproducibility and controllability, the citrate stabilization of Au-SPIONs was set as a standard procedure and only Cit-Au-SPIONs were analyzed in further experiments. Both, Cit-SPIONs and Cit-Au-SPIONs, exhibited superparamagnetic behavior, as illustrated in Figure 4a. The H_C_ values are 0.221 kA/m for Cit-SPIONs and 0.225 kA/m for Cit-Au-SPIONs. However, a large difference in their saturation magnetization was detected. After gold-coating, the saturation magnetization decreased to 40.7% from 54 to 22 Am^2^/kg.

Similar results were observed when examining the volumetric susceptibility of the samples. Relative values, referring to pure Cit-SPIONs as 100%, showed a reduction to 94% ± 2% and 89% ± 2% in Au-SPIONs and Cit-Au-SPIONs, respectively.

The X-ray diffraction (XRD) pattern of Cit-SPIONs (Figure 4b) revealed peaks at 30.2, 35.6, 43.1, 57.0 and 62.7°. The detected peaks showed accordance with characteristic peaks of magnetite and maghemite found in the literature [41,42]. The hkl values for the respective peaks were (220), (311), (400), (511) and (440). The lattice parameter and crystallite size of Cit-SPIONs was calculated with the most intense peak at 35.6°. The lattice parameter of the sample was 8.37 Å. The crystallite size was found to be about 16 nm.

The analysis of Cit-Au-SPIONs revealed the appearance of an additional gold peak at 38.1° and the broadening of the peak at 43.1°, while the characteristic peaks of Cit-SPIONs were still present. The gold lattice parameter was calculated to be 4.09 Å by using the peak at 38.1°. The gold crystallite size was determined to be 29 nm.

**Figure A4:**
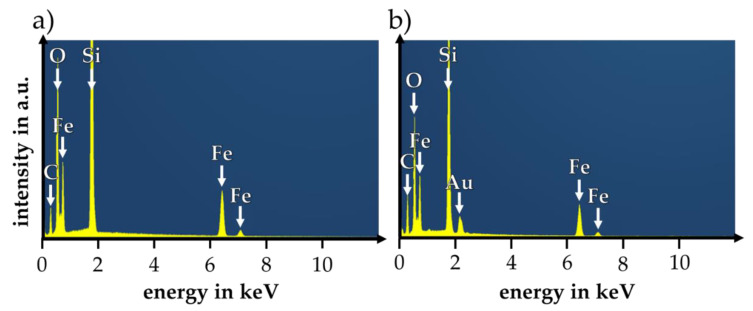
Energy dispersive X-ray spectra of (**a**) Cit-SPIONs and (**b**) Cit-Au-SPIONs. A gold related peak appeared after gold coating process.

For an additional confirmation of the gold formation on the SPION surface, energy dispersive X-ray spectroscopy (EDX) was performed on Cit-SPIONs and Cit-Au-SPIONs, as shown in Figure A4. A peak related to gold was found in the spectrum of Cit-Au-SPIONs. The Au/Fe ratio (*wt/wt*) on Cit-Au-SPIONs was calculated to be 0.23 ± 0.03.

**Figure A5:**
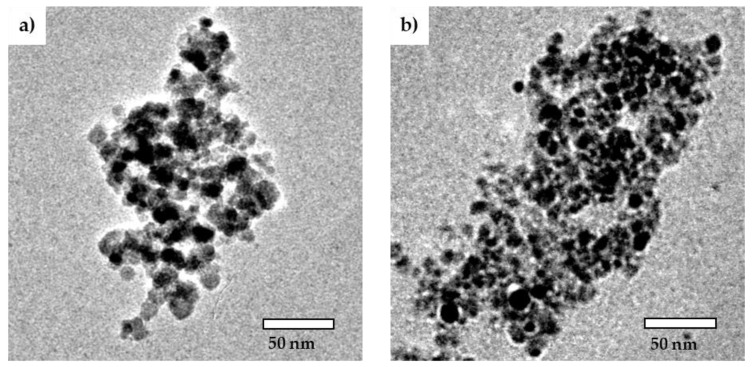
Transmission electron microscopy (TEM) images of (**a**) Cit-SPIONs and (**b**) Cit-Au-SPIONs.

Transmission electron microscopy (TEM) images of Cit-SPIONs and Cit-Au-SPIONs show aggregates of particles independent of the gold-coating, as depicted in Figure A5.

### 2.2. Investigation of Parameters Affecting Cit-Au-SPION Synthesis

#### 2.2.1. Citrate Concentration

**Figure 5:**
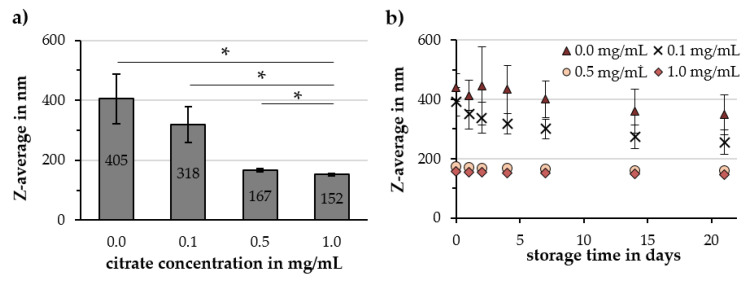
Influence of the citrate concentration in Cit-Au-SPION dispersions on (**a**) the Z-average and (**b**) the particle stability during 21 days of storage at 4 °C. Statistically significant changes are marked with * for *p* < 0.05.

**Figure A6:**
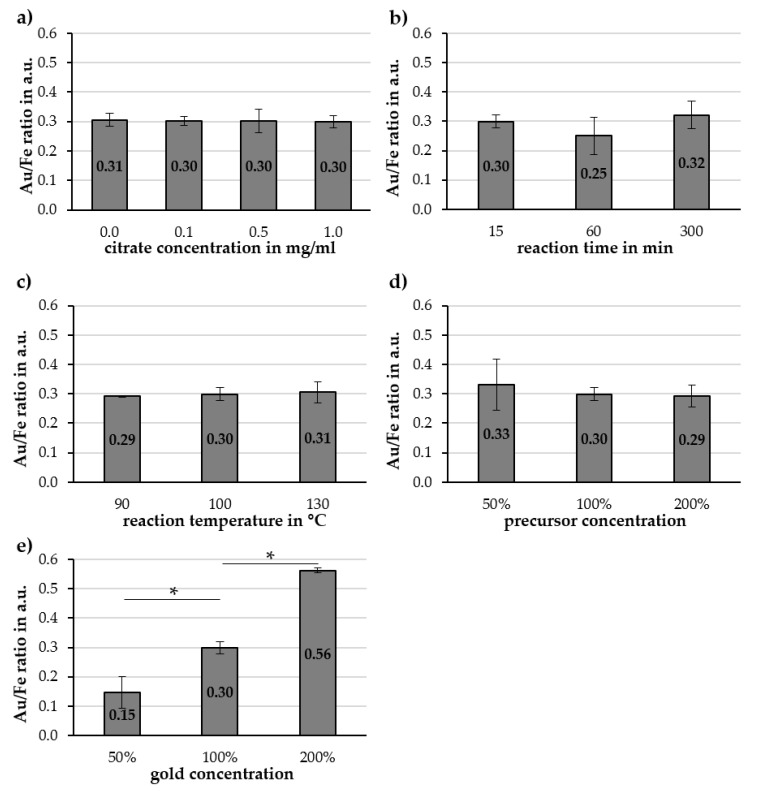
Gold to iron ratios of synthesized Cit-Au-SPIONs with varied (**a**) citrate concentration in the particle dispersion, (**b**) reaction times, (**c**) reaction temperatures, (**d**) precursor concentrations and (**e**) gold concentrations. The targeted Au/Fe ratio for (**a**–**d**) was 0.30. For (**e**) the targeted ratios were 0.15, 0.30 and 0.60 for 50%, 100% and 200%, respectively. Statistically significant changes are marked with * for *p* < 0.05.

By increasing the citrate concentration in the Cit-Au-SPION dispersion, the Z-average decreased from 405 ± 83 to 318 ± 60, 167 ± 5 and 152 ± 5 nm for 0.0, 0.1, 0.5 and 1.0 mg citrate/mL, respectively, as shown in Figure 5a. The corresponding PDI values were 0.25 ± 0.06, 0.25 ± 0.03, 0.21 ± 0.01 and 0.19 ± 0.01. With the increase in the citrate concentration, particle batches became more reproducible with less heterogeneous particle sizes and smaller deviations in the Z-average between each other as well as during the storage time over 21 days (Figure 5b). Z-averages changed only by 14 and 9 nm during 21 days for 0.5 and 1.0 mg citrate/mL, respectively. The ζ-potential at pH 7 showed no significant differences even when compared to non-stabilized Au-SPIONs. The unadjusted pH value of the particle dispersion, however, increased as more citrate was added. The pH rose from 2.89 ± 0.14 to 3.19 ± 0.25, 5.52 ± 0.17 and 6.21 ± 0.14 for 0.0, 0.1, 0.5 and 1.0 mg citrate/mL (Table 2). Volumetric susceptibilities did not reveal an influence of the citrate concentration. The Au/Fe ratio (*wt/wt*) of the particles was kept at 0.30 ± 0.04 for all samples, as shown in Figure A6.

#### 2.2.2. Reaction Time

**Figure 6:**
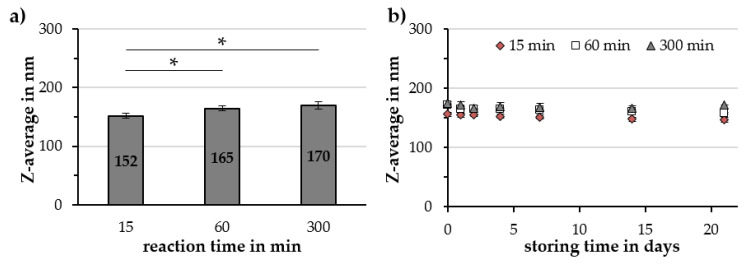
Influence of the reaction time during Cit-Au-SPION synthesis on (**a**) the Z-average and (**b**) the particle stability during 21 days of storage in a fridge. Statistically significant changes are marked with * for *p* < 0.05.

Extending the reaction time from 15 to 60 and 300 min resulted in an increased Z-average from 152 ± 5 to 165 ± 6 and 170 ± 6 nm, respectively (Figure 6a), but it did not affect the hydrodynamic size during storage (Figure 6b). No statistically significant changes in the PDI, ζ-potential, pH value and volumetric susceptibility were found as the reaction times were varied (Table 3).

#### 2.2.3. Reaction Temperature and Precursor Concentration

**Figure A7:**
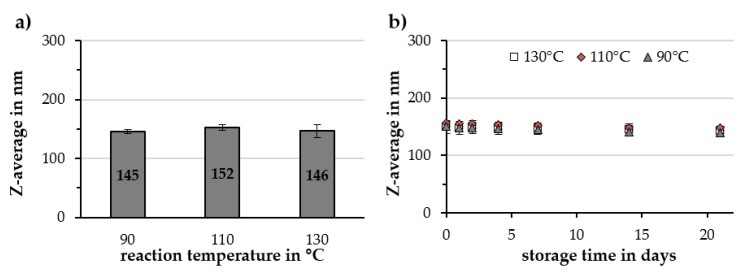
Influence of the reaction temperature measured in an oil bath surrounding the reaction flask on (**a**) the Z-average and (**b**) the particle stability during 21 days of storage in a fridge. Statistically significant changes are marked with * for *p* < 0.05.

**Figure A8:**
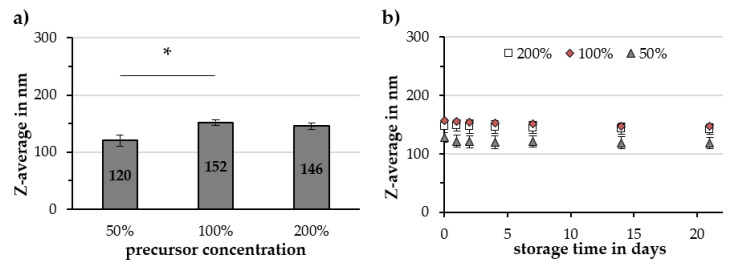
Influence of the precursor concentration (iron and gold) of Cit-Au-SPIONs on (**a**) the Z-average and (**b**) the particle stability during 21 days of storage in a fridge. Statistically significant changes are marked with * for *p* < 0.05.

By changing the reaction temperature measured in an oil bath surrounding the reaction flask from 90 to 110 and 130 °C, no statistically significant changes were found for the Z-average, PDI, ζ-potential, pH value and volumetric susceptibility (data shown in Figure A7 and Table A1). Similarly, changing the iron and gold precursor concentration together resulted in only minor changes. Upon doubling the precursor concentrations, no statistically significant changes in the Z-average, PDI, ζ-potential and volumetric susceptibility were found (Table A2). Halving the precursor concentration led to a decrease in particle size to 120 ± 10 nm (Figure A8) and in PDI value to 0.16 ± 0.01. The ζ-potential and volumetric susceptibility were unaffected by the 50% decrease in the precursor concentration. However, with increasing precursor concentrations, the pH value of the particle dispersion significantly decreased from 6.59 ± 0.13 for 50% to 6.21 ± 0.14 for 100% and 5.50 ± 0.17 for 200% iron and gold concentration.

#### 2.2.4. Gold Concentration

**Figure 7:**
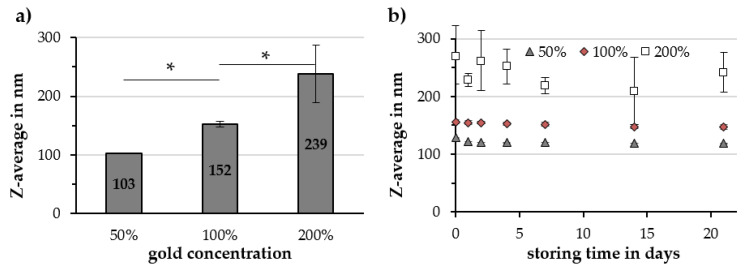
Influence of the gold concentration of Cit-Au-SPIONs on (**a**) the Z-average and (**b**) the particle stability during 21 days of storage in a fridge. Statistically significant changes are marked with * for *p* < 0.05.

Particles synthesized with 50%, 100% and 200% gold concentration showed an Au/Fe ratio of 0.15 ± 0.05, 0.30 ± 0.02 and 0.56 ± 0.01, respectively. An increase in the Z-average from 103 ± 1 to 152 ± 5 and 239 ± 49 nm for 50%, 100% and 200% gold concentration were observed, as illustrated in Figure 7a. By doubling the gold concentration, the reproducibility of the synthesis decreased and large deviations in the Z-average appeared between the particle batches and during their 21 days storage time, as depicted in Figure 7b. Halving the gold concentration resulted in a more narrow PDI value, while doubling the gold concentration revealed large deviations in the PDI. The ζ-potential and volumetric susceptibility were not influenced by the gold salt concentration, while raising the concentration of gold decreased the pH value of the particle dispersion from 6.66 ± 0.06 to 6.21 ± 0.14 and 5.05 ± 0.10 for 50%, 100% and 200% of gold, respectively (Table 4).

### 2.3. Cell Toxicity and Uptake of Cit-Au-SPIONs

Sterile Cit-Au-SPIONs were used for cell testing. The important physicochemical characteristics such as the Z-average and susceptibility of Cit-Au-SPIONs did not change after autoclaving, as shown in Table 5.

**Figure 8:**
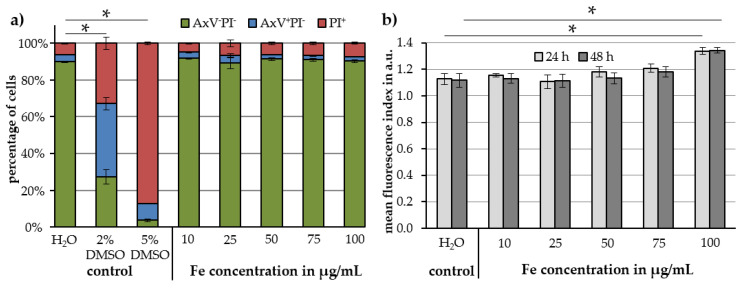
(**a**) Jurkat cell viability evaluated by Annexin A5 fluorescein isothiocyanate (FITC) conjugate (AxV)/propidium iodide (PI) staining after 48 h of incubation with different Cit-Au-SPION concentrations and (**b**) Cit-Au-SPION uptake into Jurkat cells evaluated by Lucifer Yellow staining after 24 and 48 h of incubation with different concentrations of Cit-Au-SPIONs. Statistically significant changes are marked with * for *p* < 0.05.

Jurkat cell viability results by Annexin A5 fluorescein isothiocyanate (FITC) conjugate (AxV)/propidium iodide (PI) staining are presented in Figure 8a. Viable cells (AxV^−^PI^−^) are illustrated in green, early apoptotic cells (AxV^+^PI^−^) in blue and late apoptotic or necrotic cells (PI^+^) in red. When comparing cells treated with H_2_O as a control to cells treated with different concentrations of Cit-Au-SPIONs, no statistically significant changes in cell viability were observed up to the highest Cit-Au-SPION concentration even after 48 h. At 100 µg Fe/mL, cell viability of 90% ± 1% was observed. More diluted Cit-Au-SPION dispersions exhibit viabilities of 92% ± 0.3%, 89% ± 3%, 91% ± 1% and 91% ± 1% for 10, 25, 50 and 75 μg Fe/mL, respectively.

To investigate the uptake of Cit-Au-SPIONs into the cells, Lucifer Yellow staining was performed. The resulting mean fluorescence index is displayed in Figure 8b for iron concentrations of 10, 25, 50, 75 and 100 μg/mL at incubation times of 24 and 48 h. In comparison to the H_2_O control, a significant increase in the mean fluorescence index was detected only at iron concentrations of 100 μg/mL. After 24 h, the mean fluorescence index reached values of 1.13 ± 0.04 for H_2_O and 1.34 ± 0.03 for 100 μg iron/mL. Comparable values were measured after 48 h with 1.12 ± 0.05 for H_2_O and 1.34 ± 0.02 for 100 μg iron/mL.

### 2.4. L-Cysteine Binding to Cit-Au-SPIONs

**Figure 9:**
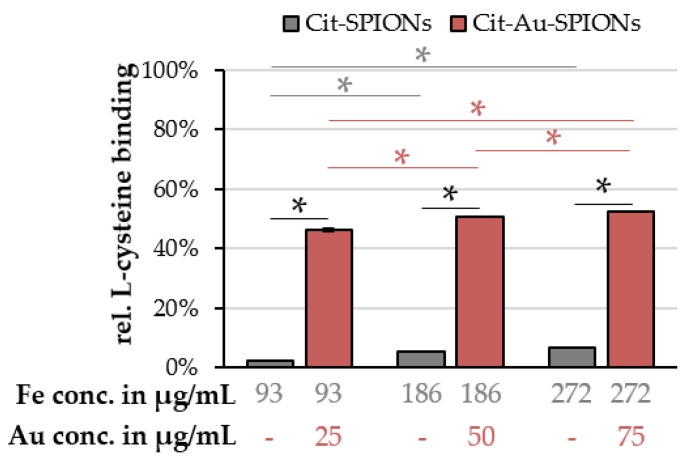
Relative L-cysteine binding to Cit-SPIONs and Cit-Au-SPIONs after incubation in a 1 mM L-cysteine solution for 2 h. Statistically significant changes are marked with * for *p* < 0.05.

Cit-SPIONs and Cit-Au-SPIONs were incubated with L-cysteine solution in order to test whether the specific binding of thiol-containing molecules to the gold surface occurs in higher amounts as compared to iron oxide surfaces. By analyzing the supernatants of Cit-SPIONs and Cit-Au-SPIONs using Ellman′s reagent after the incubation with 1 mM L-cysteine, the relative binding of L-cysteine to the particles was calculated, as depicted in Figure 9.

For Cit-SPIONs, the relative L-cysteine binding increased with higher particle concentrations from 2.3% ± 0.1% to 5.4% ± 0.4% and 6.6% ± 0.2% for concentrations of 93, 186 and 272 µg Fe/mL, respectively. Cit-Au-SPIONs exhibited significantly higher relative cysteine bindings of 46.4% ± 0.7%, 50.8% ± 0.2% and 52.6% ± 0.3% for concentrations of 93, 186 and 272 µg Fe/mL and 25, 50 and 75 µg Au/mL.

## 3. Discussion

This study aimed to create size controllable, colloidal stable, reproducible and non-cytotoxic gold-coated SPIONs that show promising properties for attaching thiol-containing molecules on their surface.

### 3.1. Cit-SPION Characterization

Mere Cit-SPIONs were synthesized following the protocol of Mühlberger et al. modified by a dialysis step instead of magnetic separation to remove excess citrate that might interfere during the gold reduction step. In comparison to SPIONs reported in that study (hydrodynamic size of 58 nm, PDI of 0.149) [43], Cit-SPIONs exhibited a larger hydrodynamic size (107 nm) and a slightly narrower size distribution (0.129). Through the dialysis, excess citrate was removed from the dispersion, which could have caused an instability of dispersed Cit-SPION cores. The small cores could have therefore aggregated onto larger clusters causing an increase in average hydrodynamic size and a decrease in PDI. The large aggregation of SPION clusters found on SEM images can be explained by some inhomogeneity during the freeze-drying sample preparation as the solvent sublimates.

### 3.2. Gold-Coating of Cit-SPIONs

During the reduction of chloroauric acid (HAuCl_4_) onto Cit-SPIONs to create Au-SPIONs, the SPION dispersions′ color turned from black to a deep red, which could indicate the formation of nanosized gold [44,45]. Non-stabilized Au-SPIONs exhibited insufficient size controllability, reproducibility and long-term stability. Thus, Au-SPIONs were additionally stabilized with citrate after the gold reduction. The resulting Cit-Au-SPIONs showed improved reproducibility, long-term stability in dispersion and moderate dispersion pH values.

During the reduction of HAuCl_4_ to solid gold, the acidic HAuCl_4_ and the stabilizing citrate on the particle surfaces are consumed and H^+^ is generated as a byproduct of the reaction [46]. The consequential decrease in pH value is especially pronounced for non-stabilized Au-SPIONs, while the addition of citrate to Cit-Au-SPIONs may buffer the generated H^+^, thus causing the observed significant increase in the dispersions′ pH value. Furthermore, the strong decrease in the pH value of Au-SPIONs could indicate that citrate ions that stabilize the Cit-SPION cores react almost completely with the added HAuCl_4_ solution. Therefore, the citrate is consumed which leaves the synthesized Au-SPIONs without surface stabilization. The resulting agglomeration of Au-SPIONs could lead to the increase in the Z-average and PDI. By adding more citrate after the gold-coating, the formed gold-coated SPIONs can be re-stabilized, which is demonstrated by more a controllable and stable Z-average, smaller PDI and higher pH value of Cit-Au-SPIONs.

FTIR measurements further provided evidence that citrate was mostly consumed on non-stabilized Au-SPIONs. The disappearance of peaks related to C-OH oscillations and the decrease in relative intensities of COO^−^ related peaks can be explained by the consumption of citrate, which has three carboxyl groups and one hydroxyl group. During gold reduction, the citrate is converted to dicarboxy acetone [44,46]. Dicarboxy acetone has two carboxyl groups, but no hydroxyl group. Thus, features related to C-OH disappear and COO^−^ peaks are less pronounced for non-stabilized Au-SPIONs. After the addition of citrate in Cit-Au-SPIONs, the intensities of these features reappear, which can indicate the stabilization with citrate.

The comparison of the magnetic behavior of pure Cit-SPIONs and Cit-Au-SPIONs revealed a decrease in the saturation magnetization to 40.7% after the gold-coating. Similarly, the volumetric susceptibility dropped by ~10% after the coating process. Both findings could be caused by the shielding of the superparamagnetic iron oxide cores with a paramagnetic gold layer, which might reduce the total magnetic behavior of Cit-Au-SPIONs. Elbialy et al. also reported a change of the magnetic properties after the gold-coating, whereby their particles decreased to 32.8% of the starting saturation magnetization after the coating process, dropping from 58 to 19 Am^2^/kg [34]. In a direct comparison with this study, citrate stabilization may have contributed to slightly enhanced saturation magnetization, which was 22 Am^2^/kg in our Cit-Au-SPIONs.

The XRD peaks detected for Cit-SPIONs and Cit-Au-SPIONs were in accordance with the characteristic peaks of magnetite and maghemite found in the literature [41]. For Cit-Au-SPIONs, two additional peaks at 38.1° and 44.5° were detected, which are correlated to characteristic gold peaks [42]. Thus, XRD data indicate the formation of crystalline gold with a lattice parameter of 4.09 Å calculated using the peak at 38.1°. This value is in line with the lattice parameter found in the literature (4.08 Å) [47]. The iron oxide lattice parameter of Cit-SPIONs was calculated using the peak at 35.6° and had a value of 8.37 Å. This value is comparable to the lattice parameters of magnetite (8.40 Å) and maghemite (8.35 Å) reported in the literature [41,48]. The crystallite size was found to be about 16 nm, which is below the critical size of 20 nm for superparamagnetic behavior in magnetite and maghemite [49,50]. Elbialy et al. reported a crystallite size of 10 nm for their similarly synthesized SPIONs [34]. The iron precursor ratio of these particles was different in comparison to Cit-SPIONs, however, which could have caused the differences in crystallite size.

The gold crystallite size was observed to be 29 nm. This value is comparable with the size of pure Au-NPs synthesized with the Turkevich method [51,52]. Hence, it could be assumed that individual Au-NPs had formed during the reduction and that these Au-NPs interacted with the SPION cores. Elbialy et al. estimated their gold shell around the SPIONs to be 10–15 nm in thickness [34]. The calculation of the crystallite size of the iron oxide core by using the (311) iron oxide peak in the Cit-Au-SPION XRD pattern, however, resulted in a value of 35 nm instead of 16 nm when calculated from the Cit-SPION pattern. The difference in the iron oxide cores′ crystallite sizes could be due to the overlap of the (311) iron oxide peak and the (111) gold peak. Further analysis, such as high-resolution transmission electron microscopy, will be necessary in the future to examine the exact structure of the gold coating.

### 3.3. Factors Influencing the Gold-Coating Process

When investigating the factors influencing the particle synthesis and coating, the citrate concentration as well as the gold concentration in the reaction liquid seem to have a major effect on the controllability of the Z-average, reproducibility and long-term stability of Cit-Au-SPIONs. While an increase in citrate concentration led to smaller hydrodynamic sizes, enhanced long-term stability, and a more neutral pH value with better reproducibility, increasing the gold concentration on SPIONs showed opposite effects. Longer reaction times resulted in an increase in the Z-average, which might be explained by the Ostwald ripening of the particles at elevated temperatures. Changing the reaction temperature did not have any effect on Cit-Au-SPIONs. Lower temperatures might have an influence, but further experiments would be necessary to test the potential effects. At 130 °C, the reaction solution started to boil. Therefore, there is no use in a further increase in the reaction temperature. Doubling the precursor concentration for iron and gold did not influence the particles except for a decrease in the pH value of the dispersion due to the increased formation of H^+^ during the gold reduction. In contrast, halving the precursor concentrations resulted in smaller hydrodynamic sizes of the particles and an increase in the pH. A strong influence was found when the Fe/Au ratio was varied. With an increase in the Au/Fe ratio, the hydrodynamic size of the particles increased while size controllability and reproducibility could not be maintained. Higher gold concentrations decreased the pH value of the particle dispersion presumably due to the higher H^+^ concentration which was generated during gold reduction.

### 3.4. Cytotoxicty and Uptake into Cells of Cit-Au-SPIONs

No statistically significant cell toxicity was found when Jurkat cells were incubated for 48 h with Cit-Au-SPION concentrations up to 100 µg Fe/mL. Cell viability was comparable between the H_2_O control and the highest concentration of Cit-Au-SPIONs (100 µg Fe/mL) after 48 h of incubation. Jurkat cells were chosen for primary experiments as they are non-adherent T cells present in blood that will have contact with the particles during a potential application. Mühlberger et al. investigated the viability of EL4 T lymphocytes from mouse lymphoma treated with non-dialyzed citrate-stabilized SPIONs. At iron concentrations of 100 μg/mL, they reported cell viabilities of 87%, 80% and >70% for time points at 0, 24 and 48 h, respectively. [43]. Although EL4 and Jurkat cells are similar cell types, a direct comparison is not possible. Nevertheless, coating SPIONs with gold might decrease the slightly toxic effects of pure SPIONs on cells. After the gold-coating, the particle size of the SPIONs increases, which reduces the probability of particle uptake into the cells and inducing harmful effects inside the cell. Furthermore, gold can be considered as a bioinert material which intrinsically shows weak interactions with biological tissue and cells [53]. Therefore, a gold shell could prevent harmful interactions of the SPION core with the cell even after internalization.

Results of Lucifer Yellow staining indicate that Cit-Au-SPIONs are incorporated into Jurkat cells only if the particle concentration is sufficiently high. Lower concentrations seemed to show no cellular uptake of the particles. A similar effect was described by Mühlberger et al. regarding their non-dialyzed citrate-stabilized SPIONs [43]. The cellular uptake of their particles in EL4 cells was observed at iron concentrations of 50 μg/mL and higher. Further experiments with higher particle concentrations and primary human cells are necessary to reach solid conclusions, however, on the toxicity and uptake of Cit-Au-SPIONs in different cell types.

### 3.5. L-Cysteine Binding

Increasing the concentration of the particles from a relative concentration of 25 to 50 and 75 µg gold/mL led to an increase in L-cysteine attachment in Cit-SPION and Cit-Au-SPION samples. As the concentration of the particles increase, there is more available surface for the L-cysteine to attach. Thus, the relative surface binding of L-cysteine increases in both samples as the particle concentration is raised. Low amounts of L-cysteine might non-specifically bind to the iron oxide surface [54] or show some interaction with the stabilizing citrate on Cit-SPIONs [55]. The amount of relative surface binding on Cit-Au-SPIONs, however, was significantly higher than the binding on pure Cit-SPIONs. The specific attraction of the thiol group of the L-cysteine to the gold surface seems to be stronger than the unspecific attraction to the iron oxide surface via the carboxylic acid or amino group. Therefore, L-cysteine could be bound to Cit-Au-SPIONs in larger amounts than to pure Cit-SPIONs. These data indicate that Cit-Au-SPIONs constitute promising candidate particles for binding thiol-containing molecules or drugs.

## 4. Materials and Methods

### 4.1. Materials

Iron (III) chloride hexa-hydrate (99%) and iron (II) chloride tetra-hydrate (99%) were purchased from Merck (Darmstadt, Germany). Ammonia (NH_3_, 25%), sodium citrate (99%), potassium bromide (KBr) (spectroscopy grade), dimethyl sulfoxide (DMSO, 99.5%), L-cysteine (98%), disodium hydrogen phosphate (99%) and Ellman′s reagent (98%) were purchased from Carl Roth (Karlsruhe, Germany). Chloroauric acid (HAuCl_4_, 99.9%), propidium iodide (PI, 94%) and Lucifer Yellow CH dipotassium salt (LY) were purchased from Sigma-Aldrich (Taufkirchen, Germany). RPMI 1640 medium, GlutaMAX supplement, Hoechst 33342 (Hoe), Annexin A5 fluorescein isothiocyanate (FITC) conjugate (AxV) and DiIC_1_(5) (1,1′-dimethyl-3,3,3′,3′-tetramethylindodicarbocyanine iodide, DiI) were purchased from Thermo Fisher (Waltham, MA, USA). Fetal calf serum (FCS) was purchased from Biochrom (Berlin, Germany). T cell leukemia cells Jurkat (ACC 282) were purchased from DSMZ (German collection of Microorganisms and Cell Cultures, Braunschweig, Germany). Ringer′s solution was purchased from Fresenius Kabi (Bad Homburg, Germany). Deionized water was produced using a Merck Milli-Q purification system. All reagents were used without further purification.

### 4.2. Synthesis of Citrate-Stabilized SPIONs (Cit-SPIONs)

Citrate-stabilized SPIONs (Cit-SPIONs) were synthesized according to the protocol described by Mühlberger et al. [43], modified by dialysis instead of magnetic separation to remove excess citrate. In a typical synthesis procedure, iron (II) and iron (III) salts were dissolved in H_2_O to achieve a solution containing 13.2 mg iron/mL. After deoxygenizing with argon, 25% NH_3 (aq)_ was rapidly injected into the vigorously stirred iron salt solution. The now blackened SPION dispersion was further stirred for 10 min at room temperature. Next, 15 mL of a 293.3 mg/mL sodium citrate solution was added to the dispersion while mixing at 400 rpm. The temperature was raised to 90 °C and the dispersion was stirred for 30 min under reflux. After cooling to room temperature, the dispersion was transferred to dialysis tubes. The dispersion was dialyzed in H_2_O for 6 h. The dialyzed particles were filtered through a 0.2 μm filter membrane and stored at 4 °C until further use. These particles are referred to as Cit-SPIONs. All particle batches were synthesized in triplicate (*n* = 3).

### 4.3. Synthesis of Gold-Coated SPIONs

Gold-coating of Cit-SPIONs was achieved by following the procedure of Elbialy et al. with some adaptations [34]. In a regular synthesis, 35 mL of a diluted Cit-SPION dispersion was added to a three-necked flask. The iron concentration of the Cit-SPION dispersion was previously adjusted using H_2_O. The dispersion was deoxygenized in argon for 5 min. The temperature was raised to 110 °C while stirring. Different amounts of a HAuCl_4_ solution were injected in one shot to the vigorously stirred SPION dispersion. The dispersion turned deeply red during stirring for 15 min at 110 °C. After cooling to room temperature, the dispersion was transferred into glass flasks. The particles are further referred to as Au-SPIONs. All particle batches were synthesized in triplicate (*n* = 3).

### 4.4. Synthesis of Citrate-Stabilized Gold-Coated SPIONs

To achieve an enhanced long-term stability and size control over Au-SPIONs, the above-mentioned procedure was extended by further stabilizing the particles using sodium citrate to create Cit-Au-SPIONs. After the addition of the HAuCl_4_ solution to the Cit-SPION dispersion and stirring for 15 min at 110 °C as described above, different amounts of a citrate solution were added. The dispersion was further stirred for 30 s and then cooled to room temperature. All particle batches were synthesized in triplicate (*n* = 3).

### 4.5. Investigation of Synthesis Parameters for Cit-Au-SPIONs

To investigate the effects of different parameters on Cit-Au-SPIONs during synthesis, the process parameters were varied. The citrate concentration in the final Cit-Au-SPION dispersion was adjusted to 0.1, 0.5 and 1.0 mg/mL and compared to non-stabilized Au-SPIONs to examine the additional stabilization with citrate. Secondly, while the citrate concentration was kept at 1.0 mg/mL, the reaction time varied from 15 to 60 and 300 min. Furthermore, the reaction temperature, measured in an oil bath surrounding the reaction flask, decreased from 110 to 90 °C and increased to 130 °C. The overall dilution of the reaction solution was adjusted to 50%, 100% and 200% iron and gold precursor concentrations in relation to the Cit-Au-SPION synthesis procedure. Lastly, only the gold concentration was modified to 50%, 100% and 200%, while the iron concentration was kept at 100% in relation to the Cit-Au-SPION synthesis procedure. All particles were synthesized in individual triplicates (*n* = 3).

### 4.6. Physicochemical Characterization

#### 4.6.1. Atomic Emission Spectroscopy (AES)

The iron content in Cit-SPIONs and the iron and gold content in Au-SPIONs and Cit-Au-SPIONs was determined by AES measurements (Agilent 4200 MP-AES, Agilent Technologies, Santa Clara, CA, USA). AES samples were prepared by dissolving Cit-SPIONs in nitric acid while Au-SPIONs and Cit-Au-SPIONs were dissolved in a 1:1 (*v/v*) mixture of nitric acid and hydrochloric acid and the dissolved particles were diluted with H_2_O.

#### 4.6.2. Scanning Electron Microscopy (SEM) and Energy Dispersive X-ray Spectroscopy (EDX)

The size and shape of Cit-SPIONs, Au-SPIONs and Cit-Au-SPIONs were determined using a scanning electron microscope (Auriga, Zeiss, Oberkochen, Germany). The samples were prepared by freeze-drying (Alpha 1–2 LD plus, Martin Christ, Osterode am Harz, Germany) previously diluted SPION dispersions for 24 h on silicon sample holders.

The EDX detector (Silicon Drift Detector (SDD)-X-MaxN, Oxford Instruments, Tubney Woods, Abingdon, United Kingdom) is part of the SEM. Therefore, the samples used in the SEM could also be used for the EDX analysis.

#### 4.6.3. Transmission Electron Microscopy (TEM)

Transmission electron microscopy (TEM) images were taken using a CM30 TEM/STEM (Philips, The Netherlands) operating at 300 kV. Specimens were prepared by drop casting diluted nanoparticle dispersions onto carbon-coated copper grids (Plano, Germany). For the CCD camera, a Tietz Fast Scan-F114 (Tietz Video and Image Processing Systems GmbH, Gauting, Germany) was used.

#### 4.6.4. Dynamic Light Scattering (DLS)

The hydrodynamic size of the SPIONs was determined by DLS using a Zetasizer Nano (Malvern instruments, Worcestershire, United Kingdom). Samples were prepared by diluting the dispersions with H_2_O. The iron concentration of Cit-SPIONs was adjusted to 50 μg/mL. The iron concentration of Au-SPIONs and Cit-Au-SPIONs was adjusted to 25 μg/mL. The measurement was conducted at 25 °C.

The long-term stability of the SPION dispersions was determined by measuring the hydrodynamic size over three weeks. Time points were set to 0, 1, 2, 4, 7, 14 and 21 days, with day 0 being measured directly after the synthesis procedure was finished.

#### 4.6.5. ζ-Potential Measurement

The ζ-potential of the SPIONs was investigated using the Zetasizer Nano (Malvern instruments, Worcestershire, United Kingdom). SPION dispersions were diluted with H_2_O to an iron concentration of 50 (Cit-SPIONs) or 25 µg/mL (Au-SPIONs and Cit-Au-SPIONs). The pH value was adjusted to 7.0 by the dropwise addition 1% hydrochloric acid or 1 M sodium hydroxide.

#### 4.6.6. Susceptibility Measurement

To verify the magnetic properties of the synthesized SPIONs, the magnetic susceptibility was investigated by using a magnetic susceptibility meter (MS2G, Bartington Instruments, Witney, UK). The SPION dispersions were diluted with H_2_O to an iron concentration of 1 mg/mL for the measurement.

#### 4.6.7. Vibrating Sample Magnetometry (VSM)

VSM measurements (Micromag 3900, Lake Shore Cryotronics, Montgomery, Westervill, OH, USA) were used to examine the saturation magnetization of Cit-SPIONs and Cit-Au-SPIONs at a field of 955 kA/m. Freeze-dried powder samples were used.

#### 4.6.8. Fourier Transform Infrared Spectroscopy (FTIR)

For the investigation of citrate stabilization and gold formation, the samples were characterized with Fourier transform infrared spectroscopy. Potassium bromide pellets containing 1% (*wt/wt*) of sample were analyzed using a FTIR spectrometer (Alpha-P, Bruker, Billerica, MA, USA). OPUS software (Bruker, Billerica, USA) was used for background subtraction and baseline correction.

#### 4.6.9. Ultraviolet–Visible Spectroscopy (UV–Vis)

The absorption spectra of aqueous Cit-SPION, Au-SPION and Cit-Au-SPION dispersions with a concentration of 25 µg Fe/mL were measured using a UV–Vis Spectrophotometer (Libra S22, Biochrom, Cambridge, United Kingdom). The measurement range was set between 250 and 700 nm with a step size of 2 nm.

#### 4.6.10. X-ray Diffraction (XRD)

To further analyze the gold formation on the SPIONs, an X-ray diffraction (MiniFlex 600, Rigaku, Tokyo, Japan) was performed. Adequate amounts of Cit-SPIONs and Cit-Au-SPIONs were freeze-dried to receive about 30 mg of particle powder. The powder was dispersed in ethanol and dripped onto a sample holder for XRD analysis. A Cu K_α1_ X-ray source with a wavelength of 1.5406 Å and a step size of 0.03°/s was used. The distance of the atomic planes d_hkl_ calculated by Bragg′s law (1) together with Equation (2) [56] was used to receive the lattice parameter *a* of the samples.

(1) $$d_{\mathrm{hkl}}\;=\;\frac{\lambda }{2\times \sin\left(\theta \right)}$$

(2) $$d_{\mathrm{hkl}}\;=\;\frac{a}{\sqrt{h^{2}+k^{2}+l^{2}}},$$

where λ is the wavelength, θ is the diffraction angle and h, k, l are the Miller indices of the diffraction plane. With the usage of the Debye-Scherrer formula (3), the crystallite size d_cry_ of the samples was calculated.

(3) $$d_{\mathrm{cry}}\;=\;\frac{0.9\;\times \;\lambda }{\mathrm{FWHM}\;\times \;\cos\left(\theta \right)},$$

where FWHM represents the full-width at half maximum value of the XRD peaks [56].

### 4.7. In Vitro Toxicity

For the investigation of the potential toxic effects of produced Cit-Au-SPIONs on the cell viability of the T cell leukemia cell line Jurkat (ACC 282), flow cytometry was used according to the protocols described by Mühlberger et al. [43]. Cit-Au-SPIONs were sterilized by autoclaving and analyzed afterwards to ensure comparable particle properties to non-sterile particles. Jurkat cells were cultured in RPMI medium supplemented with 10% fetal calf serum and 1% L-glutamine at 37 °C in a humidified 5% CO_2_ atmosphere. In total, 900 μL of a Jurkat cell suspension (1.5 × 10^5^ cells) was transferred into each well of a 24-well plate, and 100 μL of prediluted sterile Cit-Au-SPION dispersions was added to the wells. The final iron concentration in the wells was set to 10, 25, 50, 75 and 100 μg/mL. The nanoparticle-treated samples were incubated at 37 °C in a humidified 5% CO_2_ atmosphere and were analyzed at 0, 24 and 48 h after the addition of the SPIONs. Sterile H_2_O was used as a negative control, while 2% and 5% of sterile DMSO were used as positive controls for cell death.

#### 4.7.1. Cell Viability

A Hoe/AxV/PI/DiI staining solution containing 1 μL/mL Hoe of a 10 mg/mL solution, 2 μL/mL AxV, 66.7 ng/mL PI and 0.4 μL/mL DiI of a 10 μM solution in Ringer′s solution was freshly prepared at each time point. In total, 250 μL of the as prepared staining solution was added to 50 μL of a well-mixed cell sample. The stained cell sample was incubated under light protection at 4 °C for 20 min before analyzing its fluorescence with a flow cytometer (Gallios, Beckman Coulter, Krefeld, Germany).

#### 4.7.2. Particle Uptake into Cells

To investigate whether particles enter the cell or whether they are adsorbed onto the cell membrane, Lucifer Yellow staining was performed as described by Mühlberger et al. [43]. Lucifer Yellow staining was performed under light protection. In the procedure, 20 μL of a 2 mg/mL Lucifer Yellow stock solution was added to 18 mL cell suspension containing 3 × 10^6^ cells. In total, 900 μL of the stained cell suspension (1.5 × 10^5^ cells) was transferred into each of 18 wells of a 24-well plate. A total of 100 μL of prior diluted sterile Cit-Au-SPION dispersions were added to each well. The final iron concentration in the wells was set to 10, 25, 50, 75 and 100 μg/mL. The final concentration of Lucifer Yellow in each well was 2 μg/mL. The samples were incubated under light protection at 37 °C in a humidified 5% CO_2_ atmosphere.

To differentiate between aggregated particles and cells of various viability, the Lucifer Yellow stained cell samples were further stained with Hoe, PI and DiI. The Hoe/PI/DiI staining solution containing 1 μL/mL Hoe of a 10 mg/mL solution, 66.7 ng/mL PI and 0.4 μL/mL DiI of a 10 μM solution in Ringer′s solution was freshly prepared at each time point. A total of 250 μL of the as prepared staining solution was added to 50 μL of a well-mixed Lucifer Yellow-stained cell sample. The stained cell samples were incubated under light protection at 4 °C for 20 min before analyzing their fluorescence with flow cytometry.

Flow cytometry data were analyzed using the Kaluza software (Version 2.1, Beckman Coulter, Krefeld, Germany).

### 4.8. L-Cysteine Binding on Cit-Au-SPIONs

L-cysteine was dissolved in a 100 mM sodium phosphate buffer (pH 8) to generate a 10 mM L-cysteine solution. Adequate amounts of the Cit-Au-SPION dispersion were added to the sodium phosphate buffer to yield 0.9 mL of buffered Cit-Au-SPION dispersions. A total of 0.1 mL of the 10 mM L-cysteine solution was added to the 0.9 mL buffered Cit-Au-SPION dispersions to achieve samples containing 1 mM L-cysteine with Au concentrations of 25, 50 and 75 μg/mL in the total sample volume. The samples were incubated at room temperature for 2 h while being mildly mixed at 250 rpm. As a negative control, pure Cit-SPIONs were used. The concentrations of the Cit-SPION samples were adjusted to fit the exact iron concentrations of the three Cit-Au-SPION samples. After 2 h of incubation, all samples were transferred in Vivaspin^®^ tubes (100,000 MWCO) and centrifuged for 30 min at 10,000 rcf. The clear supernatants were used in the Ellman assay, which was conducted according to the manufacturers′ instructions Thermo Scientific Ellman′s reagent (Product No. 22582). A total of 250 μL of the prior made phosphate buffer was added into individual wells of a 96-well plate. Subsequently, 5 μL of a 4 mg/mL Ellman′s reagent phosphate buffer solution was added to each well, followed by the addition of 25 μL of the test samples and controls. The whole well plate was incubated for 15 min under gentle shaking. The absorption of the individual wells was analyzed at a wavelength of 412 nm using a plate reader (FilterMax F5, Molecular Devices, Biberach an der Riß, Germany).

### 4.9. Statistical Analysis

Statistical significance was examined by Student′s *t*-test with a *p*-value of 0.05. Statistical significances are marked with an asterisk.

## 5. Conclusions

This study presented a novel SPION system with thiol-binding ability as a fundamental carrier system for various medical applications, such as intravascular drug delivery or photothermal therapy. Citrate-stabilized SPION cores were coated with a gold shell by the adaptation of a simple, aqueous and moderate procedure reported in the literature. Reaction parameters such as citrate-stabilization, reaction time, reaction temperature, precursor concentration and gold concentration were varied to investigate their influences on the particle properties and to generate a reproducible and controllable nanosystem. Low cytotoxicity in Jurkat cells and an enhanced attachment of thiol-containing L-cysteine confirmed the potential of Cit-Au-SPIONs for medical applications requiring specifically designed surface functionalization.

The surface attachment of large thiol-containing molecules and the binding capacity of, e.g., drugs onto Cit-Au-SPIONs must be confirmed in future studies. Furthermore, hemocompatibility tests will be necessary to ensure that the particles have no negative impact on the whole blood and blood components. In further studies, the magnetic attraction of the SPIONs towards the targeted tissue must be likewise evaluated.

## Figures and Tables

**Table 1:** Comparison of the basic parameters of Cit-SPIONs, Au-SPIONs and Cit-Au-SPIONs.

	Z-Avg. in nm	PDI in a.u.	ζ-Potential @ pH 7 in mV	pH Value in a.u.	Relative Susceptibility in a.u.
Cit-SPIONs	107 ± 3	0.15 ± 0.03	–48.0 ± 6.3	8.27 ± 0.07	100%
Au-SPIONs	405 ± 83	0.25 ± 0.06	−43.5 ± 0.6	2.89 ± 0.14	94% ± 2%
Cit-Au-SPIONs	152 ± 5	0.19 ± 0.01	−48.6 ± 0.3	6.21 ± 0.14	89% ± 2%

Z-avg.: Z-average; PDI: polydispersity index; Cit: citrate; Au: gold; SPIONs: superparamagnetic iron oxide nanoparticles.

**Table 2:** Comparison of the basic parameters of Cit-Au-SPIONs with varied citrate concentrations.

Citrate Conc. in mg/mL	Z-Avg. in nm	PDI in a.u.	ζ-Potential @ pH 7 in mV	pH Value in a.u.	Relative Susceptibility in a.u.
0.0	405 ± 83	0.25 ± 0.06	−43.5 ± 0.6	2.89 ± 0.14	94% ± 2%
0.1	318 ± 60	0.25 ± 0.03	−44.9 ± 2.5	3.19 ± 0.25	89% ± 4%
0.5	167 ± 5	0.21 ± 0.01	−47.6 ± 2.0	5.52 ± 0.17	90% ± 2%
1.0	152 ± 5	0.19 ± 0.01	−48.6 ± 0.3	6.21 ± 0.14	89% ± 2%

Conc.: concentration; Z-avg.: Z-average; PDI: polydispersity index.

**Table 3:** Comparison of the basic parameters of Cit-Au-SPIONs with varied reaction times.

Reaction Time in min	Z-Avg. in nm	PDI in a.u.	ζ-Potential @ pH 7 in mV	pH Value in a.u.	Relative Susceptibility in a.u.
15	152 ± 5	0.19 ± 0.01	−48.6 ± 0.3	6.21 ± 0.14	89% ± 2%
60	165 ± 6	0.18 ± 0.01	−50.0 ± 0.9	6.25 ± 0.13	84% ± 3%
300	170 ± 6	0.19 ± 0.01	−48.2 ± 1.8	6.34 ± 0.19	85% ± 2%

Z-avg.: Z-average; PDI: polydispersity index.

**Table 4:** Comparison of the basic parameters of Cit-Au-SPIONs with varied gold concentrations.

Gold Concentration	Z-Avg. in nm	PDI in a.u.	ζ-Potential @ pH 7 in mV	pH Value in a.u.	Relative Susceptibility in a.u.
50%	103 ± 1	0.15 ± 0.01	−50.3 ± 0.9	6.66 ± 0.06	87% ± 9%
100%	152 ± 5	0.19 ± 0.01	−48.6 ± 0.3	6.21 ± 0.14	89% ± 2%
200%	239 ± 49	0.14 ± 0.06	−46.7 ± 1.5	5.05 ± 0.10	86% ± 3%

Z-avg.: Z-average; PDI: polydispersity index.

**Table 5:** Comparison of the Z-average and volumetric susceptibility of Cit-Au-SPIONs before and after autoclaving.

	Z-avg. in nm	PDI in a.u.	Volumetric Susceptibility ×10^−3^ in a.u.
Cit-Au-SPIONs	145 ± 2	0.17 ± 0.01	5.0 ± 0.3
Sterile Cit-Au-SPIONs	141 ± 1	0.17 ± 0.01	5.1 ± 0.1

Z-avg.: Z-average; Cit: citrate; Au: gold; SPIONs: superparamagnetic iron oxide nanoparticles.

## Appendix A

**Table A1:** Comparison of the parameters of Cit-Au-SPIONs with varied reaction temperatures.

Reaction Temp. in °C	Z-Avg. in nm	PDI in a.u.	ζ-Potential @ pH 7 in mV	pH Value in a.u.	Relative Susceptibility in a.u.
90	145 ± 4	0.18 ± 0.01	−50.8 ± 1.3	6.14 ± 0.16	87% ± 4%
110	152 ± 5	0.19 ± 0.01	−48.6 ± 0.3	6.21 ± 0.14	89% ± 2%
130	146 ± 11	0.19 ± 0.01	−46.9 ± 2.6	6.09 ± 0.20	96% ± 3%

Temp.: temperature; Z-avg.: Z-average; PDI: polydispersity index.

**Table A2:** Comparison of the parameters of Cit-Au-SPIONs with varied precursor concentrations.

Precursor Concentration	Z-Avg. in nm	PDI in a.u.	ζ-Potential @ pH 7 in mV	pH Value in a.u.	Relative Susceptibility in a.u.
50%	120 ± 10	0.16 ± 0.01	−45.9 ± 2.6	6.59 ± 0.13	93% ± 0%
100%	152 ± 5	0.19 ± 0.01	−48.6 ± 0.3	6.21 ± 0.14	89% ± 2%
200%	146 ± 6	0.17 ± 0.01	−47.8 ± 1.3	5.50 ± 0.17	93% ± 15%

Z-avg.: Z-average; PDI: polydispersity index.
